# EVM Loss: A Loss Function for Training Neural Networks in Communication Systems

**DOI:** 10.3390/s21041094

**Published:** 2021-02-05

**Authors:** Scott Stainton, Martin Johnston, Satnam Dlay, Paul Anthony Haigh

**Affiliations:** Intelligent Sensing and Communications Research Group, School of Engineering, Newcastle University, Newcastle upon Tyne NE1 7RU, UK; martin.johnston@newcastle.ac.uk (M.J.); Satnam.Dlay@newcastle.ac.uk (S.D.); paul.haigh@newcastle.ac.uk (P.A.H.)

**Keywords:** machine learning, loss function, optics, error vector magnitude, neural networks

## Abstract

Neural networks and their application in communication systems are receiving growing attention from both academia and industry. The authors note that there is a disconnect between the typical objective functions of these neural networks with regards to the context in which the neural network will eventually be deployed and evaluated. To this end, a new loss function is proposed and shown to increase the performance of neural networks when implemented in a communication system compared to previous methods. It is further shown that a ‘split complex’ approach used by many implementations can be improved via formalisation of the ‘concatenated complex’ approach described herein. Experimental results using the orthogonal frequency division multiplexing (OFDM) and spectrally efficient frequency division multiplexing (SEFDM) modulation formats with varying bandwidth compression factors over a wireless visible light communication (VLC) link validate the efficacy of the proposed method in a real system, achieving the lowest error vector magnitude (EVM), and thus bit error rate (BER), across all experiments, with a 5 dB to 10 dB improvement in the received symbols EVM overall compared to the baseline implementation, with bandwidth compressions down to 40% compared to OFDM, resulting in a spectral efficiency gain of 67%.

## 1. Introduction

Visible light communication (VLC) is an area of sustained research interest, as it can provide a supplementary spectrum to high-capacity wireless internet access networks. Operating on the visible wavelengths in the electromagnetic spectrum, VLC offers low-cost, rapid network deployment using white light-emitting diodes (LEDs) [1]. In general, LEDs are low bandwidth devices and as the demand for data is ever-increasing, methods to improve spectral efficiency are currently in high demand. Candidates to do this in VLC have either been through equalization [2], or through advanced modulation schemes [3]. Of the latter, of the most promising is spectrally efficient frequency division multiplexing (SEFDM), a modification of orthogonal frequency division multiplexing (OFDM) first proposed in 2006 by Rodrigues and Darwazeh in [4]. In SEFDM, orthogonality between subcarriers is broken by compressing the overall bandwidth at the cost of inter-carrier interference (ICI) [5], and machine learning is a promising topic to compensate for this.

The use of machine-learning and neural networks for communication systems is a topic of ever-increasing interest. One of the key aspects missing from the current state-of-the-art is domain-related methods of training and evaluating these systems within the communication system context [6]. Communication systems are typically evaluated via their bit error rate (BER) after forward error correction (FEC), however, evaluation of BER as an objective function for an optimization target is not feasible, for example when training models in machine learning. Due to this, in the literature for machine learning in communications, authors typically use the mean square error (MSE) or the related $l_{2}$ norm as a loss function to train their models [7,8]. This causes a disconnect between the objective function of the model, and the eventual metrics that will be reported when the model is deployed. Thus, it is clear that a performance gain would be obtained when the objective function of the training process is aligned with the metrics that the communication system will be evaluated against.

To this end, in this paper, we propose the exploitation of the error vector magnitude (EVM) in the training process as the objective loss function and show how it can be effectively utilized to improve the performance of symbol receivers. We will be doing this with a physical implementation using a VLC link. Since EVM is a common key performance indicator used in communication systems since it was first proposed in [9], and is closely related to the BER [10]. In a machine learning scenario, EVM is useful because it gives an error value in relation to a known ideal value, and is available instantly for any batch size used in the system, unlike the unit-less BER metric that requires vast numbers of bits to converge. The EVM requires knowledge of both the in-phase and quadrature parts of a signal, therefore to aid this method, we will also be using the ‘concatenated-complex’ approach. Whilst not the main contribution of this work, there exists mixed literature between this and the ‘split-complex’ approach [11,12], i.e., splitting a complex signal into its in-phase and quadrature components and training a separate, independent model for each, this paper will also demonstrate how including both can aid a model’s learning ability due to indirect retention of the phase information.

## 2. Materials and Methods

**Figure 1:**
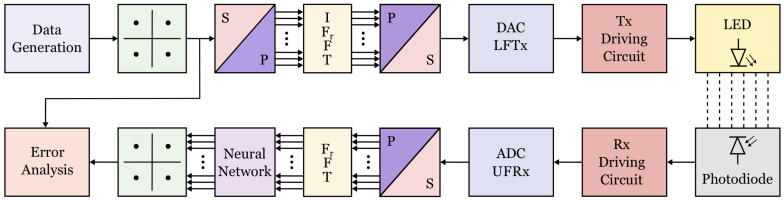
The block diagram of the system used in the experiment.

To verify the effectiveness of the EVM loss approach, we test it using two physical layer waveforms, namely orthogonal frequency division multiplexing (OFDM) and spectrally efficient frequency division multiplexing (SEFDM), with data that was obtained experimentally using a wireless visible light communication (VLC) link. It should be noted that this technique is medium-independent and was tested on a VLC link due to availability, but also works for radio links. The test setup can be found in Figure 1. $2^{16}-1$ bits were generated and mapped to the quadrature phase shift keying (QPSK) constellation. After serial-to-parallel conversion, the size of the FFT was set to generate 64 subcarriers. The symbols were then converted back from parallel to serial and transmitted over the VLC link before the inverse process was applied. The decision for placing the neural network after the FFT owes to the property of neural networks known as the ‘universal approximation theorem’ [13]. The theorem states that a neural network can learn to approximate any function, however since the FFT/IFFT pairing is already exactly known and computationally efficient, we do not incorporate this into the task of the neural network.

The experiment was conducted with NI USRP-RIOs and as such were controlled via LabVIEW software. The neural network was implemented in Python, and the final analysis into the EVM results was done in MATLAB. This work, without loss of generality, will not consider FEC and uses hard decision decoding. As EVM is inversely proportional to the square of the signal-to-noise ratio (SNR), the methodology proposed here of lowering the EVM when used before FEC will allow for exploitation of higher SNR within a soft decision decoder. OFDM is a well known and researched multi-carrier modulation scheme, whereas SEFDM is a more recently proposed non-orthogonal signal, first introduced in [4]. SEFDM employs the fractional Fourier transform (FrFT) as opposed to the standard Fourier transform (FFT) in OFDM. The use of the FrFT compresses the input signal in the frequency domain beyond the orthogonality limit of 1/T, where *T* is the symbol period. Whilst this improves spectral efficiency, it also introduces deterministic inter-carrier interference (ICI) which is detrimental to the recoverability of the signals on each subcarrier. SEFDM can be described mathematically as shown in Equation (1) [14]:

(1) $$x\left(t\right)=\frac{1}{\sqrt{N}}\sum_{m=-\infty }^{\infty }\sum_{n=0}^{N-1}s_{m,n}\exp\left[\frac{j2\pi nm\alpha }{N}\right].$$

where $s_{m,n}$ is the complex-valued symbol on the $m^{\mathrm{th}}$ SEFDM symbol on the $n^{\mathrm{th}}$ subcarrier. *N* is the total number of subcarriers and α is the bandwidth compression factor, which defines the frequency spacing of the subcarriers. In the OFDM case, α=1 and the subcarrier spacing is set to multiples of 1/T, where *T* is the symbol period. For further information including detailed mathematics of the signal, refer to [14,15].

The deterministic ICI can be modelled as a correlation matrix C as shown in Equation (2) [15]:

(2) $$\begin{array}{cc} C_{\left(l,n\right)} & =\left\{\begin{array}{cc} 1, & l=n\\ \frac{1}{N}\left[\frac{1-\exp\left[j2\pi \left(l-n\right)\right]}{1-\exp\left[\frac{j2\pi (l-n)}{N}\right]}\right], & l\neq n \end{array}\right. \end{array}$$

where *l* and *n* represent two subcarriers indices to be compared. C reduces to an identity matrix for OFDM.

**Figure 2:**
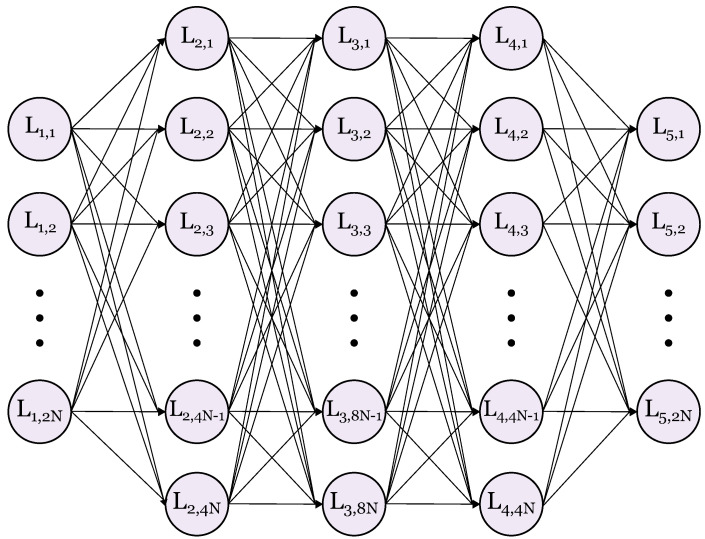
Block diagram of the neural network.

Neural networks are able to generalize any input-output sequence given sufficient neurons in the hidden layers [16]. In principle, this means that given a sufficient signal-to-noise ratio, the neural network should be able to estimate C, for any value of α. In this work, we test α≥0.6, meaning a bandwidth saving up to 40%. The neural network is designed to be trained on sequences of received QPSK symbols after transmission over a visible light link using SEFDM with a given bandwidth compression factor, or OFDM where α=1. The VLC link is characterized in [17] with a bandwidth of >50 MHz. We set our signal bandwidth to be 1 MHz to ensure a flat-band response since this paper is not about improving data rates. The signal was dc biased at 90 mA with an ac signal voltage of 2 Vpp, i.e., keeping within the linear operating region of the LED. A generalized diagram of the model used in this work can be seen in Figure 2.

The neural network input layer contains 2N neurons, which allows for separation and concatenation of the real and imaginary components of *N* subcarriers. The network requires sufficient neurons in its hidden layers to learn the complexities of the ICI and transmission channel, thus, inspired from [7], we opt for 3 hidden layers with 4N, 8N and 4N neurons respectively. Each hidden layer uses the LeakyReLU activation function with the final output layer using a linear activation, and the model was trained with the Nadam optimizer. The initial learning rate was set to $10^{-3}$ with a reduction of factor $10^{-1}$ when the validation loss did not decrease over a 5-epoch period. The received symbols are fed through the network until they reach the output layer, which also contains 2N neurons, mirroring the input. The estimate of the originally transmitted QPSK symbols are then obtained by combining the real and imaginary outputs. The EVM loss function will be introduced separately, replacing the typical mean square error which we are comparing against which is given as [18]:

(3) $$L=\frac{1}{2N}\sum_{i=1}^{2N}{\left(y_{i}-{\hat{y}}_{i}\right)}^{2}.$$

where *N* in is the number of subcarriers, $y_{i}$ is the ground truth value and ${\hat{y}}_{i}$ is the predicted value.

**Figure 3:**
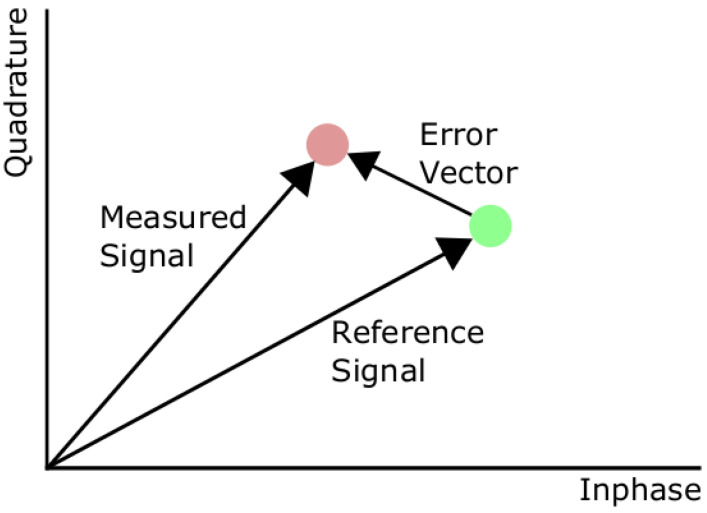
Illustration of error vector magnitude (EVM).

EVM encompasses the effects of both magnitude and phase distortions [19] and represents the difference between a measured signal and a reference signal. This is illustrated in Figure 3. Mathematically, we define the error vector $E_{k}$ for the $k^{\mathrm{th}}$ input as:

(4) $$E_{k}={\left(I_{k}-{\hat{I}}_{k}\right)}^{2}+{\left(Q_{k}-{\hat{Q}}_{k}\right)}^{2},$$

where $I_{k}$ and $Q_{k}$ are the $k^{\mathrm{th}}$ ideal in-phase and quadrature component respectively, and ${\hat{I}}_{k}$ and ${\hat{Q}}_{k}$ are the measured versions. This allows for the calculation of the EVM as [20]:

(5) $$E=\sqrt{\frac{\frac{1}{N}\sum_{k=1}^{N}E_{k}}{\frac{1}{N}\sum_{k=1}^{N}\left(I_{k}^{2}+Q_{k}^{2}\right)}}.$$

We incorporate this into the loss function when training the neural network receiver, instead of the mean square error. As is clear from the above, this requires the loss function to have knowledge of both the real and imaginary constituents of the input signal, therefore the design of the neural network has to take this into consideration. This is a simple modification that requires increasing the number of neurons of the input layer by a factor of 2. Whilst this does increase the complexity of the individual network, the overall complexity is maintained by the fact only a single network is required, rather than each one for in-phase and quadrature signal components. A second advantage of doing this is by enabling the neural network to observe both signal components, it is better able to learn how to recover the signal. This is because there is additional co-information embedded in the Cartesian form of the constellation without splitting, such as combined phase information that can lead to a more accurate joint recovery of symbols. If two neural networks are trained separately for each signal component, this information would remain unknown to the other and hence performance deteriorates comparatively.

## 3. Results

We test the proposed method with three cases, starting with an MSE loss on a split-complex network as a baseline. We then extend this to show a gain of utilizing concatenated-complex networks, and then the further gain of implementing the proposed ‘EVM loss’ system.

**Figure 4:**
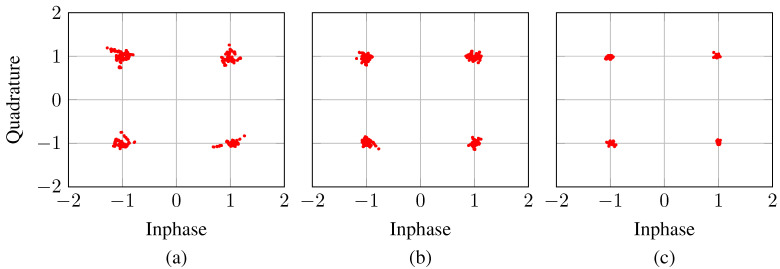
Received constellations after neural network processing for (**a**) mean square error (MSE) loss w/Split Complex, (**b**) MSE loss w/Concatenated Complex, (**c**) EVM loss w/Concatenated Complex for spectrally efficient frequency division multiplexing (SEFDM) with α=0.6.

Figure 4 shows the received constellations for these three tested approaches. The results show notable reductions in variance as one first converts from a split-complex network to a concatenated-complex network, owing to the extra indirect phase information being retained within the network. A further notable gain is then seen when converting from MSE loss in the concatenated-complex network to the proposed EVM loss, demonstrating the effectiveness of the approach.

**Figure 5:**
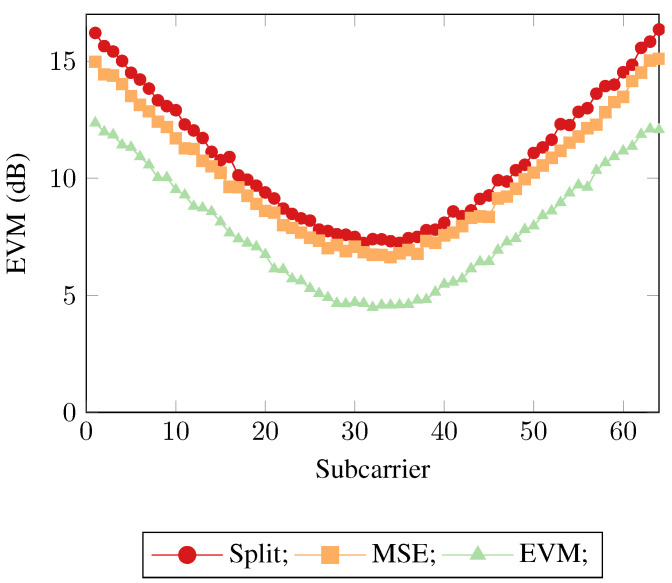
Error vector magnitudes across the subcarriers for the three neural network receiver approaches for orthogonal frequency division multiplexing (OFDM).

The first test was performed for the OFDM data transmitted over the VLC link. As can be observed from Figure 5, the highest EVM was reported by the split-complex approach. The reason for this was eluded to previously, by creating a neural network for both the real and imaginary constituents of the input signal individually, one deprives the system of information that is embedded in the relationship between the two components of the input signal. Communication system channels that affect the phase of the signal being transmitted alter this relationship, which, when divided into real and imaginary signals and trained separately is not taken into consideration. This is rectified by modifying a single neural network to allow for both the real and imaginary constituents as input. The result of this allows the neural network to exploit the phase relationship during training, meaning a lower overall error vector magnitude. Finally, the lowest error vector magnitude calculated across all subcarriers is achieved when the neural network uses the proposed EVM loss method as the objective function rather than the typical mean square error.

**Figure 6:**
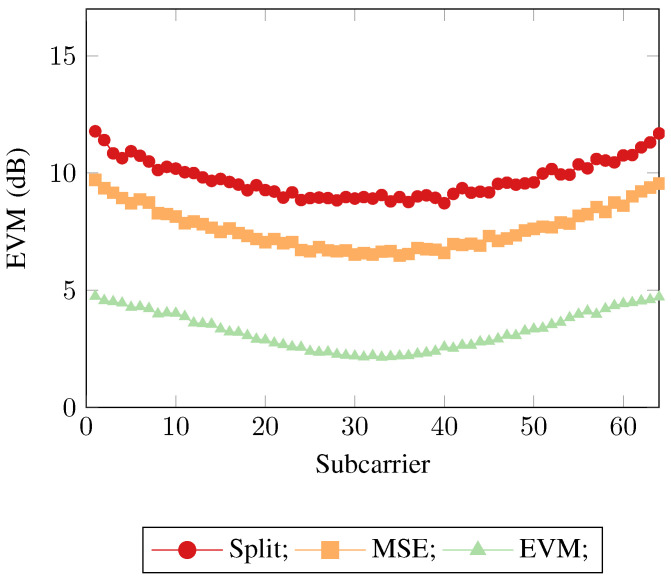
Error Vector Magnitudes across the subcarriers for the 3 neural network receiver approaches for SEFDM with α=0.8.

**Figure 7:**
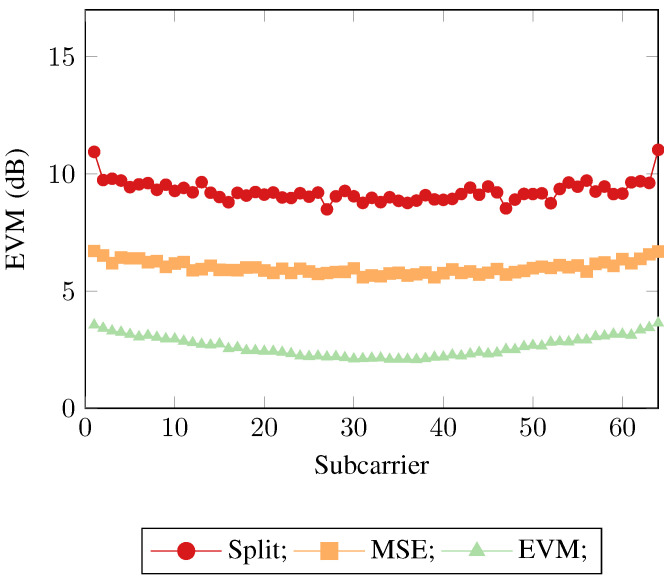
Error vector magnitudes across the subcarriers for the 3 neural network receiver approaches for SEFDM with α=0.6.

This demonstrates that the mean square error is not an optimal objective function when training neural networks in a communication systems context. Whilst the two other neural networks have minimized their mean square error objective functions as optimally as possible, the fact still remains that when being used in a communication system the training metric and the evaluation metric no longer align, leading to a degradation of the overall system performance. This can be seen when considering the implication of the implementation of the EVM loss function. The MSE loss function calculates a per neuron MSE loss, where the neuron has implicit access to the available information from its real/imaginary counterpart via the hidden layer neurons, thus the real/imaginary neurons are loosely coupled. The EVM loss function also calculates a per neuron loss, however as well as the implicit information from the network, the loss function itself explicitly examines the available information from its real/imaginary counterpart, forcing a tight coupling. These same trends can again be seen in Figure 6 and Figure 7, where SEFDM was evaluated with α=0.8 and α=0.6 being used as the transmitted physical layer waveform respectively.

## 4. Conclusions

This work has demonstrated the shortcomings of two of the typical approaches used in the implementation of neural networks in communication systems, namely; the split complex approach, whereby transmitted signals are split into their real and imaginary constituents and used to train two separate neural network models, and the use of mean square error as an objective function. It was shown that by splitting the signals into their real and imaginary components, the system was being deprived of valuable information that can be exploited and used to more accurately recover the signals. Extending this, it was also shown that there is a disconnect between training a neural network using the mean square error objective function when the communication system will be deployed and evaluated via its BER performance. This was rectified via the use of the proposed EVM loss method which seeks to reconnect the disparity between the evaluation performed when training a neural network and the evaluation of the overall communication system. The results validated the capabilities of our method, which consistently obtained the lowest EVM when deployed in a real experimental setup using OFDM and SEFDM with varying bandwidth compression factors, resulting in a 5 dB to 10 dB improvement in the received symbols measured EVM compared to the baseline implementation, with bandwidth compressions down to 40% compared to OFDM, resulting in a spectral efficiency gain of 67%.

## Data Availability

Data sharing is not applicable to this article.
